# The impact of neutrophil extracellular trap from patients with systemic lupus erythematosus on the viability, CD11b expression and oxidative burst of healthy neutrophils

**DOI:** 10.1186/s12865-021-00402-2

**Published:** 2021-02-05

**Authors:** Alimohammad Fatemi, Razieh Alipour, Hossein Khanahmad, Fereshteh Alsahebfosul, Alireza Andalib, Abbasali Pourazar

**Affiliations:** 1 Department of Internal Medicine, School of Medicine, Isfahan University of Medical Sciences, Isfahan, Iran; 2 Department of Immunology, Medical School, Isfahan University of Medical Sciences, Hezar Jerib Street, Isfahan, IR 81746-73695 Iran; 3 Department of Genetics and Molecular Biology, School of Medicine, Pediatric Inherited Diseases Research Center, Research Institute for Primordial Prevention of Non-communicable Disease, Isfahan University of Medical Sciences, Isfahan, Iran

**Keywords:** Inflammation, Damage-associated molecular patterns (DAMP), Neutrophil extracellular trap (NET), Neutrophils, Systemic lupus erythematosus (SLE)

## Abstract

**Background:**

NET (neutrophil extracellular trap) has been shown to directly influence inflammation; in SLE (systemic lupus erythematosus), it is reportedly a plausible cause for the broken self-tolerance that contributes to this pathology. Meanwhile, the role of NET is not easily explicable, and there is a serious discrepancy in the role of NET in SLE pathology and generally inflammation; in particular, the interactions of neutrophils with NET have been rarely inspected. This study evaluates the effect of NET on neutrophils in the context of SLE. The neutrophils were incubated by the collected NET (from SLE patients and healthy controls) and their expression of an activation marker, viability and oxidative burst ability were measured.

**Results:**

The level of cell mortality, CD11b expression and the oxidative burst capacity were elevated in NET-treated neutrophils. Also, the elevation caused by the SLE NET was higher than that produced by the healthy NET.

**Conclusion:**

The decreased neutrophil viability was not due to the increase in apoptosis; rather, it was because of the augmentation of other inflammatory cell-death modes. The upregulation of CD11b implies that NET causes neutrophils to more actively contribute to inflammation. The increased oxidative burst capacity of neutrophils can play a double role in inflammation. Overall, the effects induced by NET on neutrophils help prolong inflammation; accordingly, the NET collected from SLE patients is stronger than the NET from healthy individuals.

**Supplementary Information:**

The online version contains supplementary material available at 10.1186/s12865-021-00402-2.

## Background

Systemic Lupus Erythematosus (SLE) is an auto-inflammatory disorder that, like most other chronic inflammatory diseases, has a complex etiology and pathology [1]. In SLE, accelerated cell death together with the ineffective clearance of the subsequent debris, the augmented oxidative stress and their added effects result in the accumulation of myriad DAMPs (damage-associated molecular patterns), which activate the PRRs (pattern recognition receptors) on multiple innate immune cells [2, 3]. The result is a systemic inflammatory response that also culminates in the activation of adaptive immunity and eventually breaks the self-tolerance [1, 4]. As such, many autoreactive T and B lymphocytes emerge against cellular constituents, especially nuclear components, which are widespread in this disease [2]. Accordingly, the expression of pathogenic antinuclear antibodies (ANAs) is an important feature of SLE [1, 2]. The binding of these autoantibodies to their target forms immune complexes (ICs) that further amplify the systemic inflammation that can affect almost all tissues and organs of the body, thus resulting in broad clinical manifestations [1].

Because of the emerging aspects of neutrophil biology, the role of neutrophils in the onset and persistence of DAMP-induced inflammation in SLE has recently become the focus of attention [5]. Neutrophils may undergo profound cell death and supplement the burden of cell death DAMPs [2]. Neutrophils possess the enzyme complex NADPH oxidase (NOX2), the key producer of reactive oxygen species (ROS) during the oxidative/respiratory burst process that has a robust antimicrobial function [5]. Alternatively, the overproduction of ROS has been implicated in SLE pathogenesis by enhancing oxidative stress [3]. Furthermore, activated neutrophils undertake a form of inflammatory cell death called NETosis, in which they release nuclear chromatin together with granule proteins to form an extracellular web-like structure known as neutrophil extracellular traps (NETs) [6]. Most of the contributing components of NET (dsDNA, nucleosomes, and histones) act as DAMP [7]. Interestingly, the production of NET is increased in many chronic diseases [8] and the NET from these patients has more various DAMPs compared to the NET from healthy people [9]. Many studies have demonstrated that NET plays a critical role in SLE pathology [10]. They have shown that NET can be the target of autoantibodies, resulting in pathologic ICs during SLE [2]. The discovery of a subgroup of inflammatory neutrophils with an enhanced propensity to NET formation and the observation of delayed NET degradation in the subset of SLE patients reinforced previous findings [6, 11]. Further studies, however, showed that the role of NET in SLE cannot be easily explained and remains a controversial issue at the moment [6, 10].

To the researchers’ knowledge, unlike other inflammatory cells such as macrophages [12], monocytes [13], and lymphocytes [14], no studies have yet investigated the various neutrophil functions after exposure to NET. Notably, neutrophils express many PRRs whose ligands are found in NET, and the activation of these PRRs on other cells by NET has previously been reported in other studies [9, 15]. This study thus hypothesized that, as the primary responders during inflammation [8], neutrophils are influenced by NET too. To test this hypothesis and also help understand the enigmatic role of NET in SLE, the effect of NET on neutrophils was evaluated in the context of SLE with an emphasis on neutrophil functions that are important in the pathogenesis of SLE. For this purpose, NET was isolated from SLE patients and healthy individuals and its effect on the activation, apoptosis and oxidative burst ability of neutrophils was measured.

## Results

### Patients and controls

Seventeen patients who fulfilled the American College of Rheumatology (ACR) classification criteria for SLE [16] and registered at a lupus clinic affiliated with Isafahan University of Medical Sciences, and 17 healthy volunteers were enrolled in this study (Supplementary Document 1, Table S1).

### NET visualization, collection and comparison

**Fig. 1:**
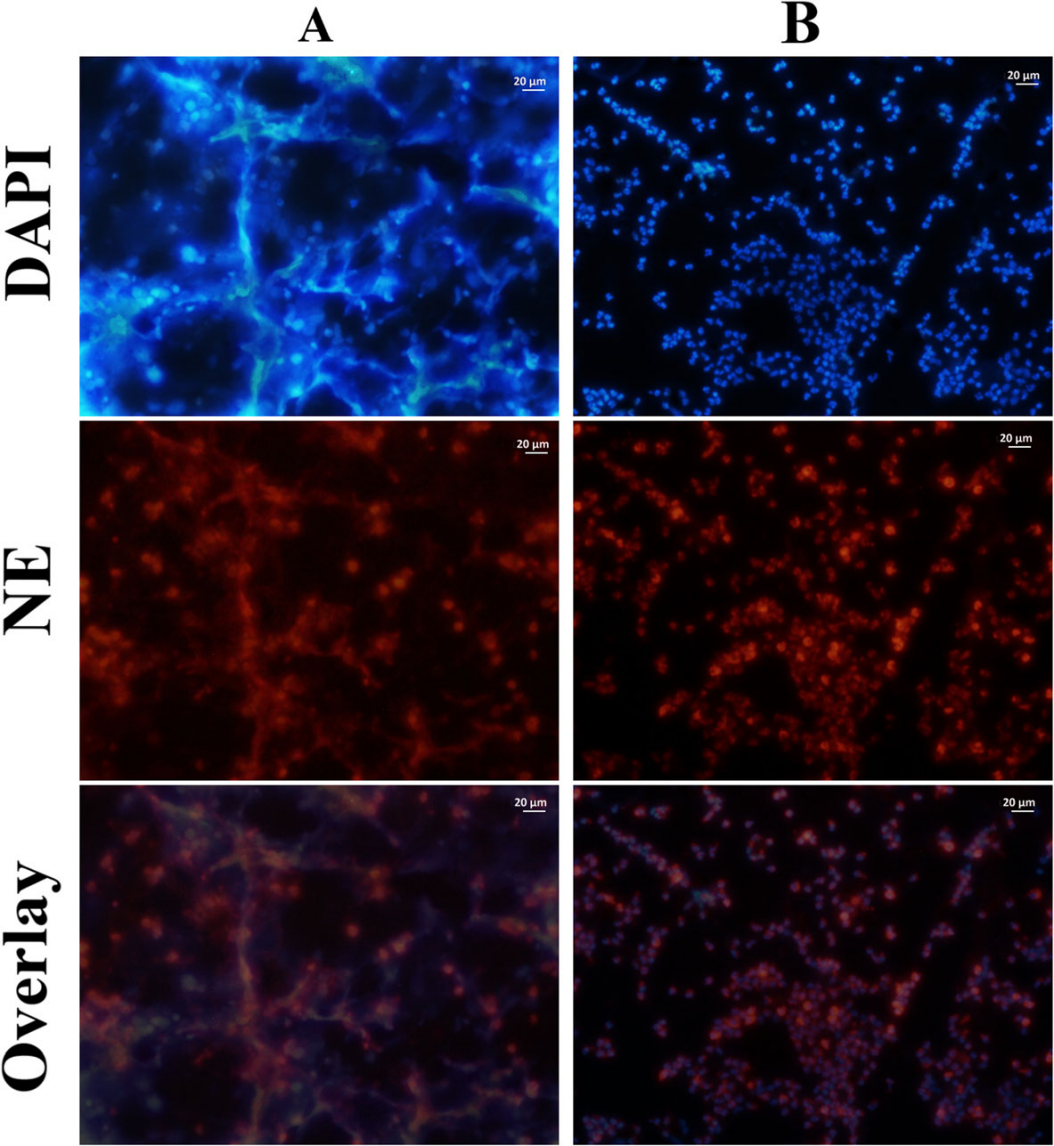
The detection of neutrophil-derived NET by immunofluorescence microscopy. To observe NET, activated (A photographs) PMNs or resting PMNs (B photographs) were fixed and immune-stained with specific anti-NE antibody followed by a fluorescent secondary antibody (red, middle photographs). The samples were stained with DAPI to counter stain the nucleus and extracellular DNA in blue (upper photographs). The overlay of the two channels is shown in the photographs at the bottom. All photos presented neutrophils from a control sample. NE: neutrophil elastase

 After PMA (Phorbol 12-myristate 13-acetate) stimulation, the production of NET from the neutrophils was confirmed with the observation of the filamentous appearance of unwound chromatin, which coexisted with neutrophil elastase (NE) (Fig. 1).

After NET collection by a non-enzymatic approach [17–19], the DNA content of the isolated NET samples was observed in a gel; also, the extent of NET production along with the protein and DNA content of NET samples were quantified and compared between patients and controls (Supplementary Document 2).

### The viability of neutrophils

**Fig. 2:**
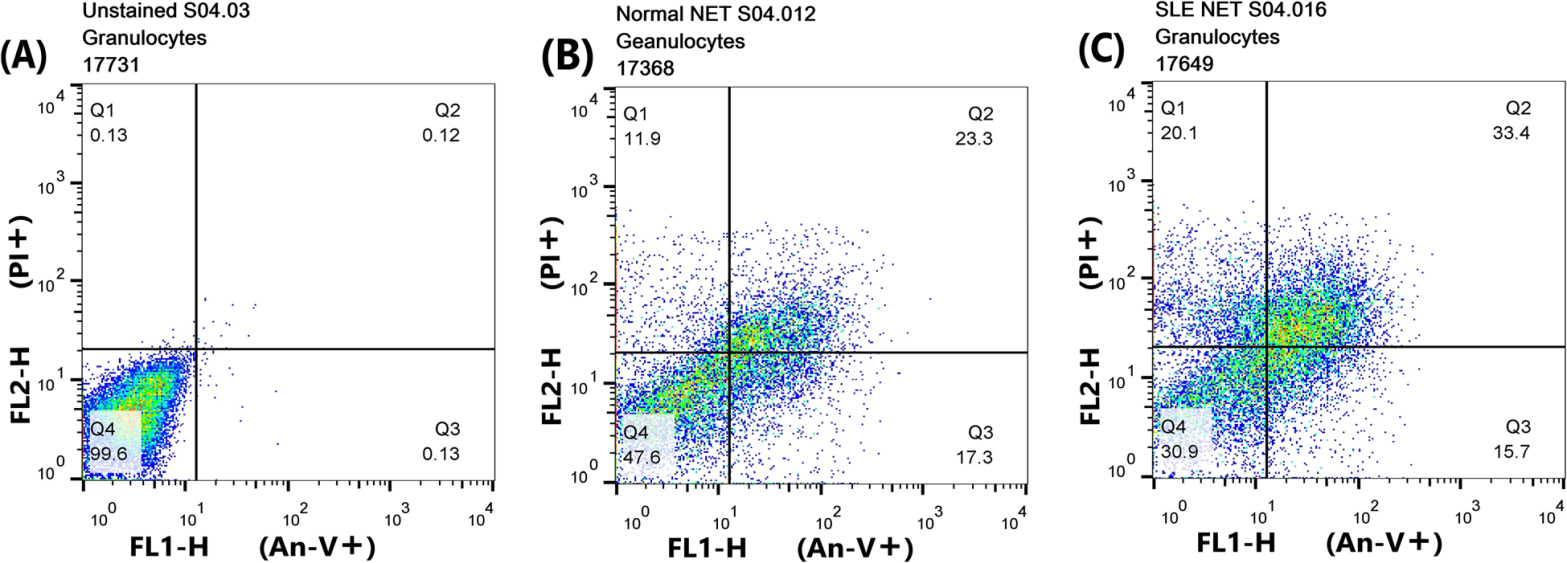
The Detection of dead cells by flow cytometry. The blood granulocytes isolated were cultured with the NET of either the healthy subjects or the patients for about 4 h, then stained with FITC-annexin-V and PI to differentiate between the viable, necrotic, early and late apoptotic cells. Plots B and C represent the same sample double-stained after coculture with normal (**b**) and SLE neutrophil-derived NET (**c**); plot A shows the same sample unstained. *An-V*: annexin-V, *PI*: Propidium Iodide

FITC-annexin-V, which binds specifically to phosphatidylserine (PS), was used to identify the early apoptotic cells. The neutrophils were also co-stained by Propidium Iodide (PI), a nucleophilic dye excluded from cells with an intact membrane to differentiate viable (annexin−/PI-), necrotic (annexin−/PI+), early (annexin+/PI-) and late (annexin+/PI+) apoptotic cells from each other (Fig. 2). The results showed a remarkable reduction in the viability of neutrophils cocultured with NET rather than those cultured alone; however, there was no statistically significant difference between the percentage of live neutrophils cocultured with NET from the healthy persons and the SLE patients (Table 1).

**Table 1:** The viability of granulocytes in contact with SLE or healthy neutrophil-derived NET

Group	Viable Cells (percent)	*P*_*value*_
SLE NETs	45.67 ± 2.38	0.101
Normal NETs	55.04 ± 1.52	
No NETs	71.26 ± 1.60	0.645
SLE NCM	71.22 ± 1.61	
Normal NCM	75.64 ± 1.51	
*P*_*value*_	0.00	

*NCM* NET control medium, which refers to the medium that was collected from unstimulated (SLE and healthy) neutrophils in NET-inducing experiments.

**Fig. 3:**
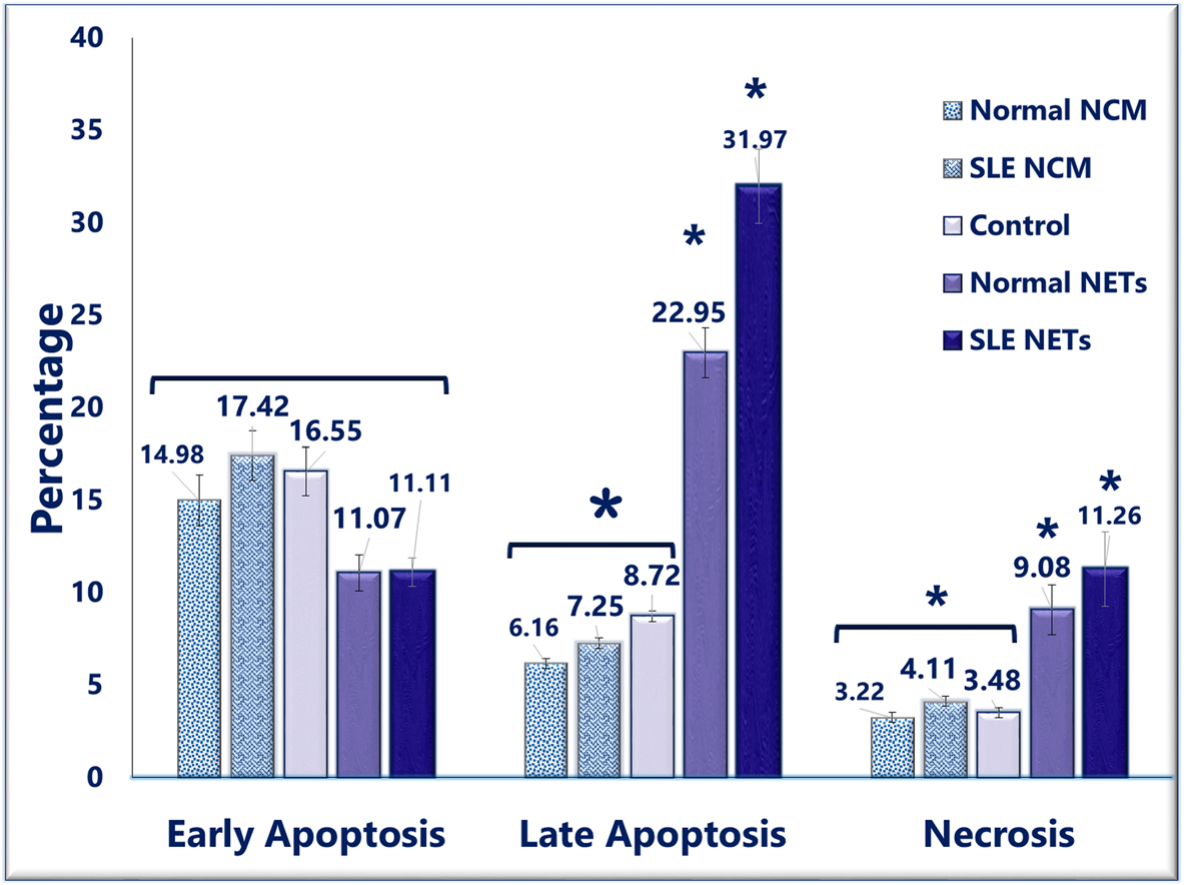
The effects of NET on the death of granulocytes. The death of neutrophils was evaluated after 4 h-contact with the NET of both healthy subjects and patients. In the case of early apoptosis, no significant differences were observed between the coculture with NET, NCM or nothing. Regarding late apoptosis, a significant increase was detected in the cocultures with either normal or SLE NET in comparison to the control cocultures (the cocultures contained no NET or NCM). The level of late apoptosis was also significantly higher in the cocultures of SLE NET than the cocultures of normal NET. The percentage of necrotic neutrophils after contact with the NET of both healthy subjects and patients for about 4 h increased significantly compared to the neutrophils in contact with no NET. The difference in the percentage of necrotic cells was statistically insignificant between the SLE NET-contained cocultures and the normal NET-contained cocultures. NCM: NET control medium, which refers to the medium that was collected from the unstimulated healthy and SLE neutrophils in the NET-inducing experiments

Analyzing the viability data in detail showed that the percentage of early apoptotic cells did not differ between the groups of neutrophils that were cultured with NET from the normal control and the patients and the no-NET cases, including the cultures with no add-in and the cultures that contained NCM (NET control medium)- which was the collected medium from the unstimulated healthy and SLE neutrophils in the NET-inducing experiments (*P* = 0.059). The percentage of late apoptotic and necrotic cells in the neutrophils cocultured with normal NET was higher compared to the neutrophils cocultured without NET (for late apoptosis, *P* = 0.000 and for necrosis, *P* = 0.032), and the percentage was highest in the neutrophils cocultured with SLE NET (for late apoptosis, *P* = 0.000 and for necrosis, *P* = 0.001). The difference between neutrophils cocultured with normal NET and those cocultured with SLE NET were statistically significant regarding late apoptosis (*P* = 0.008) and not significant regarding necrosis (*P* = 0.120) (Fig. 3).

### CD11b expression of neutrophils

**Fig. 4:**
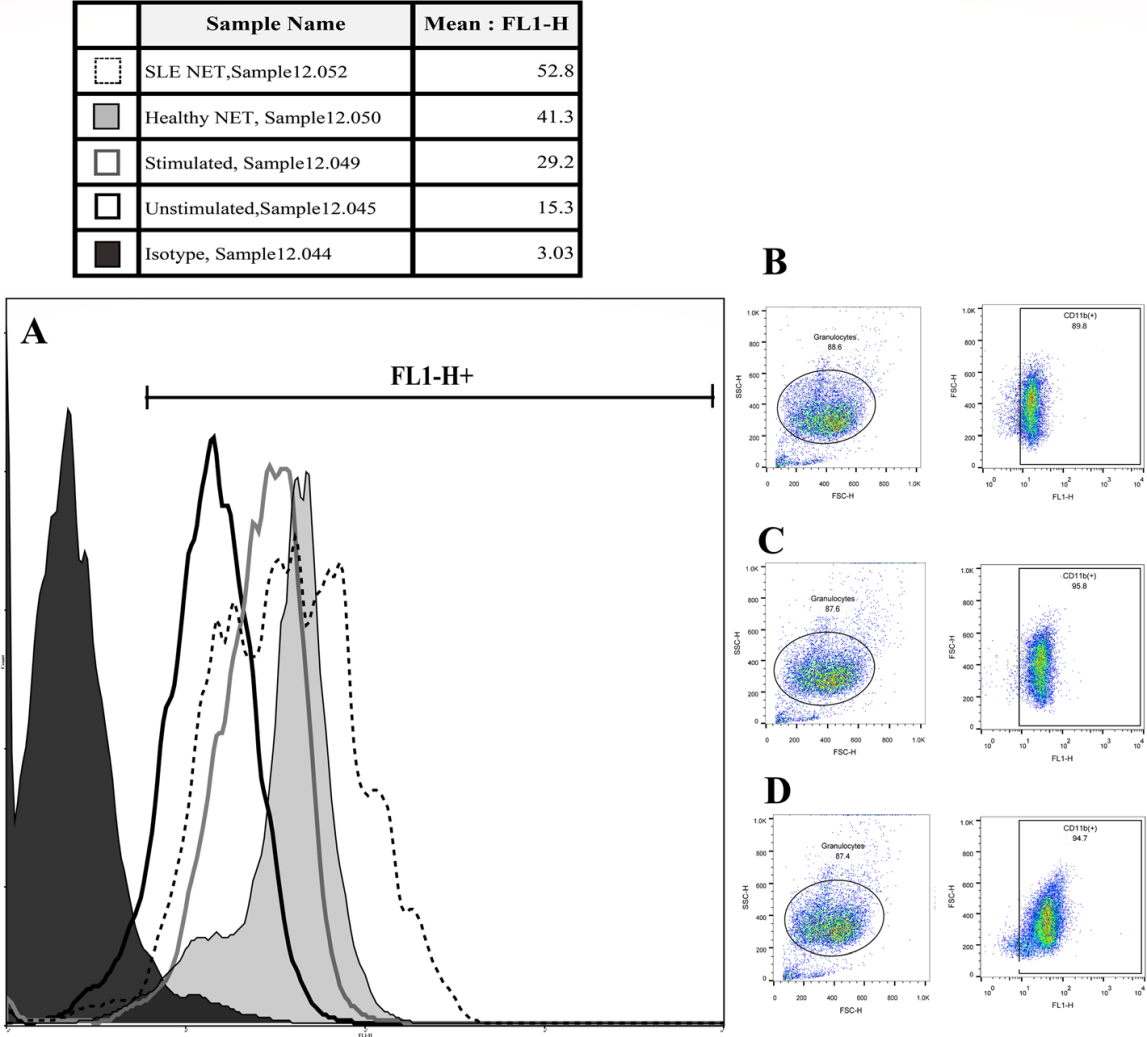
The upregulated CD11b expression following exposure to NET. Plot A shows the overlaid histogram of the unstimulated (as the negative control) and the LPS-stimulated neutrophils plus the normal-NET-treated and SLE-NET-treated LPS-stimulated neutrophils of the same sample, and their corresponding MFIs (Mean Fluorescence Intensities) are also presented in the table above the figure. The isotype control histogram is also shown in black. Plots B-D represent the unstimulated, LPS-stimulated, and normal NET-treated neutrophils of the same sample

**Fig. 5:**
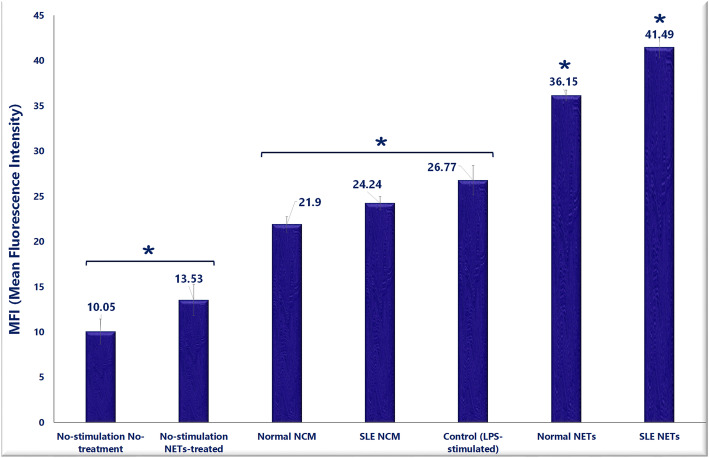
The impact of NET on the activation of granulocytes. After the designated treatments (with or without healthy and SLE NET), the upregulation of CD11b expression on the cell surface of LPS-challenged neutrophils was compared. Although the differences between the untreated and either the patient or healthy NET-treated neutrophils were significant, the difference between CD11b expression in untreated and NCM-treated granulocytes was not significant. NET pretreatment alone (without LPS stimulation) did not change the CD11b expression on the neutrophils. NCM: NET control medium; refers to the collected medium from the unstimulated healthy and SLE neutrophils in the NET-inducing experiments. MFI: Mean Fluorescence Intensity

This study showed a significant elevation in CD11b expression on the LPS-stimulated neutrophils incubated with either healthy (*P =* 0.000) or SLE NET (*P* = 0.017) compared to the untreated neutrophils (Fig. 4). Also, the neutrophils cultured with NET obtained from the SLE neutrophils showed a higher increase in CD11b expression than those cultured with NET obtained from normal neutrophils (*P* = 0.001). Yet, the differences between the cells cocultured with or without SLE and healthy NCM (or NET control medium which was collected from the unstimulated healthy and SLE neutrophils in the NET-inducing experiments) were not significant (*P* = 0.847). The pretreatment of granulocytes by SLE/healthy NET alone (without LPS stimulation) did not change the CD11b expression on the cells (*P =* 0. 491) (Fig. 5).

### Respiratory burst rate of neutrophils

**Fig. 6:**
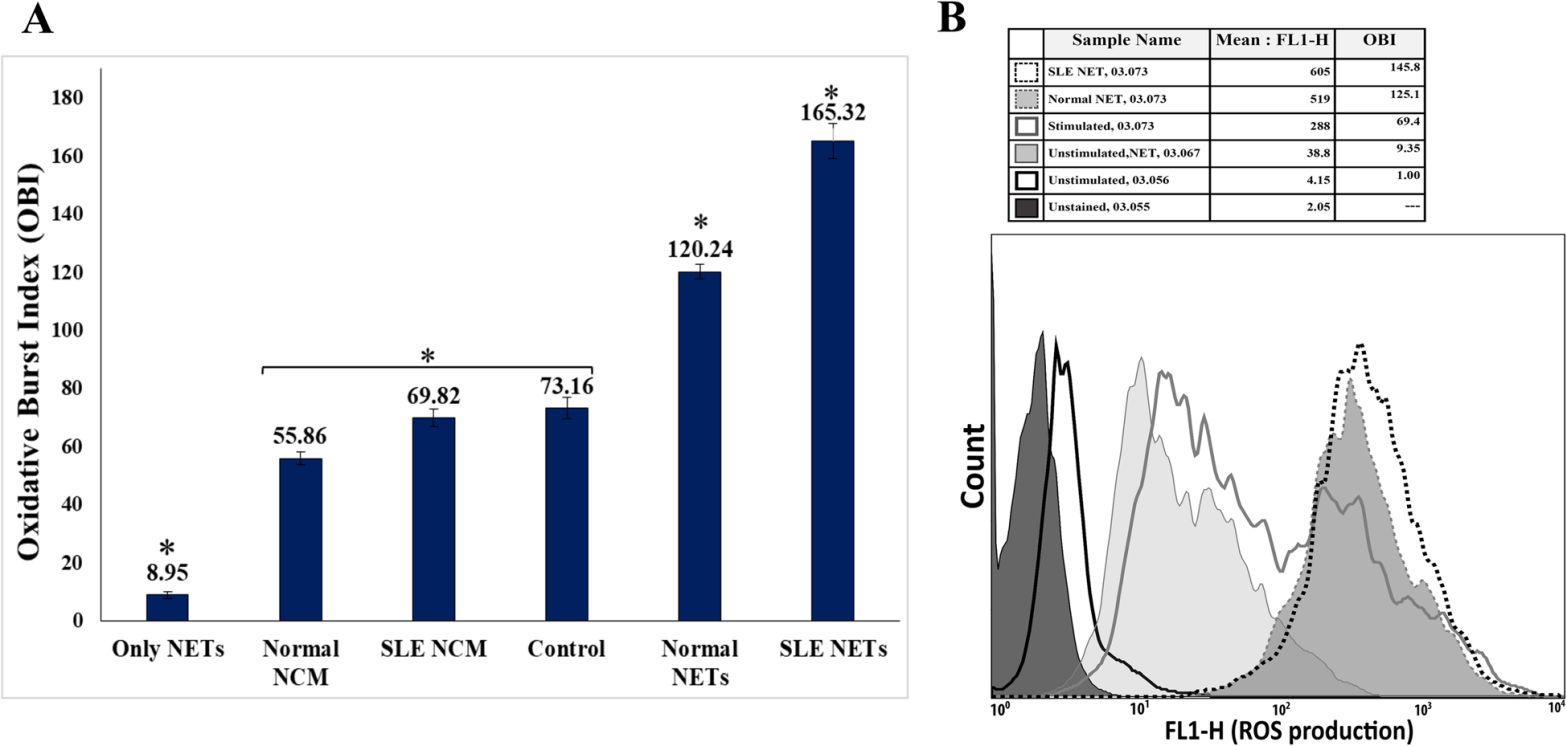
The effect of NET on the respiratory burst of neutrophils. **a** Following treatments with or without NET, PMA-stimulated granulocytes were stained by DHR and run on the flow cytometer to determine their OB capacity. The OB ability of the incubated neutrophils with either healthy and SLE NET was enhanced compared to the unincubated cells; the difference between the the two latter groups was also significant. The OB capacity of neutrophils did not change with NET pretreatment alone (with no stimulation). **b** The mean fluorescence of the activated neutrophils stained by DHR (details in the Methods section) increased because of ROS production as a result of OB. The plot shows the overlaid histograms of the unstimulated, PMA-stimulated, NET-treated unstimulated, normal-NET-treated PMA-stimulated, and SLE-NET-treated PMA-stimulated neutrophils from the same sample plus their corresponding OBI (Oxidative Burst Index) in the table. The unstained neutrophils are also shown. NCM or NET control medium refers to the medium that was collected from the unstimulated healthy and SLE neutrophils in the NET-inducing experiments

Following the discussed treatments, oxidative burst (OB) was measured in the PMA-induced neutrophils after staining with DHR. The mean fluorescence of the neutrophils indicated the amount of ROS production. The results were shown as oxidative burst index (OBI), which is the ratio of the mean fluorescence of the stimulated neutrophils to the mean fluorescence of the unstimulated neutrophils. A significant increase was detected in the respiratory burst rate of the neutrophils upon exposure to both SLE (*P* = 0.000) and healthy NET (*P* = 0.008) compared to the non-exposed cells. Besides, the PMNs incubated with SLE-neutrophil-isolated NET showed a greater increment than those incubated with the healthy neutrophil-isolated NET (*P =* 0.000). No statistically significant differences were observed between the unincubated cells and the neutrophils pre-incubated with either SLE or healthy NCM (*P* = 0.303). The pretreatment of granulocytes by SLE/healthy NET alone (without PMA stimulation) did not change the cells’ level of oxidative burst noticeably (Fig. 6).

## Disscussion

In 2004, a definition was provided for NET that was followed by a massive wave of research on neutrophils that led to emphasizing their importance in acute and chronic inflammation [5, 8]. These studies proved that NET production increases under inflammatory conditions, including in SLE [8]. NET was shown to directly influence inflammation [20] and provide autoantigens for pathologic autoantibodies during SLE [2]. More notably, as a large complex compound in which many types of DAMPs interact with each other, NET can interplay with the immune cells [12–14], including its producers, i.e. neutrophils [21]. Nonetheless, there is a serious discrepancy in the role of NET in inflammation [6, 20, 22]; in particular, the interactions of neutrophils with NET have been rarely inspected. The present study evaluated the effect of NET on neutrophils in the context of SLE.

Regarding the prominence of cell mortality in SLE [2], the viability and apoptosis of neutrophils upon encounter with normal and SLE neutrophil-derived NET were assessed; while NET augmented the percentage of late apoptotic cells, it did not affect the early apoptotic cells of neutrophils. Although some studies have reported that NET or its components induce apoptosis in some cell types [23], it cannot be deduced from the present findings that NET-treated neutrophils undergo apoptosis more than unaffected granulocytes because of the unchanged rate of early apoptosis.

To explain the increase in late apoptotic neutrophils without a raise in the early apoptotic cells, it should be noted that every dying cell with asymmetric and permeable plasma membrane that represents the annexin^**+**^/PI^**+**^ phenotype after annexin/PI double staining is not necessarily a late apoptotic cell. The externalization of phosphatidylserine (PS) and permeabilization of the plasma membrane are also seen in other types of cell death, particularly in pyroptosis and NETosis [24]. It is thus highly likely that some of the non-apoptotic dying cells, especially NETting and pyroptotic cells, are wrongly considered as the late apoptotic cells in studies that evaluated PS expression plus the plasma membrane integrity to assess apoptosis; and, as such, in the present study [25]. From this point of view, the results of the current study suggest that NET increases cell demise by the augmentation of cell death modes other than apoptosis –more likely, pyroptosis and NETosis. This conclusion clearly needs further proof by well-designed studies; however, there is already ample evidence for this deduction:

First, the percentage of PS-externalizing neutrophils with an intact membrane (annexin^**+**^/PI^**−**^), which are considered specific features of (early) apoptotic cells [24], was not affected by NET. Second, studies have reported an enhanced NETosis in human neutrophils by HMGB1 and extracellular histones, which collectively constitute more than 70% of NET-associated proteins [6, 26], and also by NET-containing media collected from PMA-stimulated neutrophil cultures [21, 27]. Third, several studies on different DAMPs, such as HMGB1 [28] and histone [29] and even the whole NET [29], have established that these DAMPs can induce pyroptosis in different types of cells [30, 31]. Similarly, Kahlenberg et al. [15] showed that the NET isolated from both the lupus and control subjects was an effective activator of pyroptosis in human macrophages. More relevantly, Chen et al. [32] demonstrated that the NET collected from PMA-stimulated PMN following coculture with normal bone marrow-derived macrophages caused cell pyroptosis. Nevertheless, neither of these or other studies have explored pyroptosis in human granulocytes during SLE, but the contribution of the DAMP-induced pyroptosis of different types of cells in the pathology of SLE has been demonstrated in a large number of reports [15, 33]; therefore, the increase in the pyroptosis of granulocytes in response to the DAMP carrier structure of NET is well conceivable. Fourth, the NET obtained from SLE patients, which is expected to carry a higher level of DAMP [9], resulted in a greater increase in the annexin+/PI+ cell population than the NET obtained from healthy subjects.

The present findings also showed a rise in the necrosis of NET-treated neutrophils, which is compatible with previous studies that had demonstrated that various DAMPs, including nucleosomes, histone, and entire NET, can induce necrosis in multiple types of immune or non-immune cells [21, 29, 34]. Also, the greater increase in the percentage of necrotic neutrophils following incubation with SLE NET compared to those incubated with healthy NET can be explained by the higher content of DAMPs in SLE NET [9, 35].

Given our results and other above-mentioned studies, the decreased number of viable neutrophils observed in the current study after 4 h of co-incubation with NET is not due to the increased apoptosis, which is considered the only kind of cell death of an anti-inflammatory nature [33], but reflects the augmentation of necrosis and potentially other inflammatory cell-death modalities, most likely, NETosis and pyroptosis [25]. Of course, this notion should be confirmed by further studies that would directly measure other types of cell death.

The upregulation of CD11b expression in neutrophils following NET exposure was assessed as an activation/degranulation marker of granulocytes [8]. The present findings revealed an obvious increase in the expression of CD11b on LPS-stimulated granulocytes after encounter with NET. The review of literature did not yield any studies on the effect of whole NET on CD11b expression on granulocytes; however, studies on separate DAMPs have shown an upregulation of CD11b on neutrophils after stimulation by nucleosome, Myeloperoxidase (MPO) and Calprotectin (S100A8 and S100A9) [36–38] –all of which are important constituents of NET [7, 26]. Moreover, an additional increase was observed following incubation with SLE NET compared to those incubated with healthy NET, which is in agreement with the reports by Ribon et al. [39], who showed a higher upregulated CD11b expression on neutrophils following exposure to higher levels of purified chromatin, or the study by Lindau et al. [40], who demonstrated a clear dose-dependent effect for nucleosomes on the upregulation of CD11b expression in neutrophils.

Despite the sufficient number of studies on the contribution of ROS and their induced oxidative stress in the pathology of SLE [1, 3], the impact of whole NET or its many well-known contributing DAMPs, such as histones, nucleosome, and chromatin, on granulocytes’ oxidative burst (OB) remains exceptionally unexplored. Likewise, few investigations were found on the effect of NET-related DAMPs on the OB of neutrophils. Sroussi et al. [41] showed that Calprotectin (S100A8 and S100A9) inhibited the OB of neutrophils. Lau et al. [36] found that MPO increases the ability of OB in human neutrophils. Tadié et al. [42] observed that the exposure of human neutrophils to HMGB1 diminishes the rate of OB. All of these DAMPs are fundamental elements of NET [6, 26]; this fact, along with the lack of data about other NET-associated DAMPs, make explaining the impact of NET on the respiratory burst of neutrophils a very intricate matter. Nonetheless, Tadié et al. [42] found the salient point that two distinct DAMPs (HMGB1 and S100B, both key components of NET) binding to the same PRR inversely affect the rate of neutrophil OB. Moreover, the same study [42] showed that different DAMPs interfere with each other’s effect on the OB of neutrophils. Similar functions also can be expected from NET as a rich source of DAMPs; although each contributing component of NET may affect the OB ability of neutrophils individually, the overall effect derives from the whole existing DAMPs in NET, and their interfering with each other is to potentiate the OB capability of neutrophils, as shown here.

One of the limitations of the present research was that the exact components of the two NET types were not compared. Thus, the responsible agent/s for more enhancements of cell mortality, CD11b expression, and OB ability of neutrophils in SLE NET could not be identified. The work of Bruschi et al. [43], who recently investigated differences between the protein composition of SLE and normal neutrophil-derived NET, may help propose scientific suggestions in this regard. They demonstrated a notable increase in the level of HMGB1 and histone H1, a slight increase in Calprotectin, and a notable decrease in the level of MPO in SLE NET compared to normal NET. Although these changes may be the causes of additional increment of mortality [27, 31] and CD11b expression [37, 44], they do not explain the increased OB capacity of neutrophils. They also found several proteins that were differently expressed by the two NET types, the impacts of which on neutrophil functions have not been elucidated yet. Regarding these issues plus the fact that the interaction of DAMPs with each other can change their effects [42, 45], determining the exact component(s) which mediate the extra effect of SLE NET need to be explored in the future, well-designed inhibitory investigations.

This study was conducted to investigate how, as a biological product enriched by multiple DAMPs [7], NET influences neutrophil effector responses and whether the NET produced by the neutrophils of SLE patients differs from healthy neutrophil-derived NET in terms of affecting neutrophil activities. According to the present findings, NET diminished the longevity of neutrophils and hastened their death. While this phenomenon may help alleviate ongoing inflammation by the elimination of neutrophils as important inflammatory cells [5, 6], the pro-inflammatory death (necrosis and potentially pyroptosis and NETosis) induced by NET can further intensify the inflammation. Moreover, it increases the burden of dead cells and thus exacerbates the disease.

Furthermore, DAMPs incorporated within NET cause neutrophils to increase CD11b upregulation. Regarding the key role of CD11b in most inflammatory activities of granulocytes, such as degranulation, neutrophil recruitment, and aggregation at inflammatory sites [8], these neutrophils can more actively contribute to the ongoing inflammation and aggravate the disease. However, recent researches suggest two anti-inflammatory functions for CD11b in neutrophils during SLE [46].

Since ROS can act as a double-edged sword in SLE pathogenesis [35], the effects observed for NET on the respiratory burst of neutrophils cannot be easily interpreted. Surplus ROS production can result in enhanced oxidative stress, which is involved in SLE pathogenesis [3]. Alternatively, the deficiency of ROS allows for the excessive degranulation of neutrophils following stimulation, leading to tissue damage and thereby sustaining inflammation via the release of DAMPs; also, the impairment of ROS can increase predisposition to SLE [35, 47].

## Conclusions

To conclude, this study indicates that, as a biological product generated under inflammatory conditions, NET can influence the effector functions of human neutrophils. The NET-induced effects on neutrophil activities seem to be more in favor of prolongation/augmentation of the inflammation rather than its diminishing, and the NET obtained from SLE patients is more potent in this effect compared to the NET obtained from healthy individuals.

## Methods

All methods and protocols were performed in accordance with the guidelines of the Ethics Committee of Isfahan University of Medical Sciences.

### Blood samples

Venous blood was collected from each of the subjects (patients and volunteers) and poured into polypropylene tubes containing EDTA-ACD (Acid Citrate Dextrose).

### Neutrophil isolation

Neutrophil isolation from the blood was carried out as previously described [48]. Briefly, after PRP (Platelet-Rich Plasma) separation and RBC sedimentation by dextran, the sample was decanted into a discontinuous two-layer density gradient of Percoll (Santa Cruz; 86 and 55%) and centrifuged at 480 rcf for 17 min at 18 °C (with no brakes). The granulocyte layer (on Percoll 86%) was then carefully removed, washed once with RPMI (5 min, 300 rcf, 18 °C) and suspended in RPMI medium. Hypo-osmotic lysis was used to lyse the residual RBCs in some of the samples. The cell viability and count were evaluated by Trypan blue exclusion. The purity of the neutrophil population was > 95% [48].

### NET formation, visualization and collection

The isolated neutrophils were seeded on poly-L-lysine-treated circle coverslips at a concentration of 10^6^ neutrophils/mL in RPMI-1640 without serum. The Cell Activation Cocktail (Biolegend) was added to the test cultures while nothing was added to the control cultures. The cells were incubated for 4 h at 37 °C in CO2 5% with 90% humidity. Shortly afterwards, the neutrophils were fixed (4% paraformaldehyde, 4 h, room temperature (RT)), permeabilized (0.5% Triton X-100, 1 min, RT) and blocked by 5% BSA (30 min, 37 °C). The coverslips were then incubated with primary anti-neutrophil elastase antibody (NP57, Santa Cruz; 1 h, 37 °C), followed by secondary m-IgGκ BP-CFL 555 antibody (Santa Cruz; 1 h, 37 °C), and were then counter stained by DAPI (Santa Cruz) to detect DNA. The specimens were visualized under the microscope (Nikon Ti-U Inverted Fluorescence Microscope).

To collect NET, we applied the method successfully used by many other researchers lately [17–19] and best described by Najmeh et al. [17], which is a simple non-enzymatic approach of NET isolation. Briefly, neutrophils were stimulated as described, and after incubation, the cells were washed by a pre-warmed medium twice with great caution. The NET was then separated from the cells by pipetting in RPMI and the NET-contained medium was collected and centrifuged (10 min at 300 rcf, RT) to precipitate the remaining cells or debris, and the cell-free supernatant was finally collected as NET. The same collection procedure was carried out for the neutrophils without stimulation and the final supernatants were collected as the NET control medium (NCM) and later used as suitable controls. For greater conformation, the DNA content of the isolated NET samples was observed in a gel (Fig. S2). Also, the amount of NET was quantified and compared between patients and controls (Table S3 and Table S4).

### Treatment with NET

To assess the impact of NET on neutrophils, the isolated neutrophils from three healthy volunteers were cultured in RPMI medium, supplemented by autologous plasma (AP) 10% at a density of 1 × 10^6^ cell/ml, and the collected NET was added at a concentration of 25% (v/v) of the culture medium. The same culture procedure was also performed on the controls (Fig. S1). All the cultures were incubated at 37 °C, in CO2 5%, with 90% humidity, for 4 h and were done in triplicate.

### Cell viability and apoptosis measurement

Once the incubation process was complete, the neutrophils were harvested, washed and resuspended in RPMI without serum and stained using an Annexin V-FITC Apoptosis detection kit (BMS500FI/300 CE, eBioscience) as per the manufacturer’s protocol and were then analyzed immediately by flow cytometry.

### Measurement of oxidative burst

After the completion of the incubation time, the neutrophil cultures were either activated or not (for the controls) by Cell Activation Cocktail (Biolegend; 1 μL to 1 × 10^6^ cell/ml) and incubated for 20 min (37 °C, CO2 5%, 90% humidity). Dihydrorhodamine 123 (DHR, Santa Cruz) was then added and the neutrophils were re-incubated for another 20 min (37 °C, CO2 5%, 90% humidity). Thereupon, the cells were placed in an ice bath (10 min), washed with cold PBS (300 g, 3 min, RT), suspended in FACS solution (flow cytometry sheath fluid) containing formaldehyde 0.5% and analyzed by flow cytometry.

### CD11b expression assay

After incubation, the neutrophils were washed, resuspended in PBS, stimulated (though not for the controls) with 100 ng/ml of endotoxin (LPS from *Escherichia coli*, serotype 0111: B4, Sigma) and incubated for 30 min at 37 °C, in CO2 5%, and with 90% humidity. The cells were then transferred to RT and either FITC anti-human CD11b mAb (ICRF44, Biolegend) or an isotype control antibody was added. The samples were incubated at RT for 20 min before they were washed twice with PBS containing BSA 0.5% (300 g, 3 min, RT) and then run on flow cytometer.

### Statistical analysis

Data were analyzed using IBM SPSS statistics 25 software. The results were reported as mean ± Standard Error (SE). The one-way ANOVA, followed by Bonferroni’s multiple comparison test, were used to compare the differences between the groups. A *p*-value of < 0.05 was considered significant.

## Supplementary Information

**Additional file 1. Supplementary Document 1.**

**Additional file 2. Supplementary Document  2.**

**Additional file 3: Fig. S1** A schematic diagram of the neutrophil culture. The schematic diagram of the treatment of neutrophils is shown in the figure in black and the subsequent stimulation related to “CD11b expression assay” is presented. Number (7) denotes the baseline expression. Numbers (6) and (5) are controls for the NET isolation; they determine whether the observed effects of the collected NET are really from NET. Number (4) determines whether NET (a mixture of the patient and healthy NET) can change CD11b expression by itself. NCM: NETs’ control medium.

**Additional file 4: Fig. S2** The visualization of DNA of NET in gel. Lane 1 corresponds to the molecular weight marker, with a higher band of 3000 bp and a lower 100-bp band. The high molecular weight bands in lanes 3, 4, 7, and 8 correspond to DNA present in NET samples. Two NCM (NET control medium) samples -collected from unstimulated neutrophils in NET-inducing experiments- were loaded in lanes 5 and 6. No sample was loaded in lane 2.

**Additional file 5: Fig. S3** A standard curve and its corresponding brief data plotted using StepOne™ software. The number of NETting neutrophils (or the copy number of the target genomic sequence) in 10 samples (5 SLE patients and 5 controls) is shown. With the standard curve generated by data from the standard dilution series, the software determined the absolute quantity for each sample. In the plot, red squares correspond to standards, and blue squares correspond to samples (all tests were performed in triplicate). The quantity of a sample refers to the absolute copy number of the target gene (here, *TLR-4*) in the sample, which was taken as the number of neutrophils that released their nuclear DNA (or the absolute count of NETting neutrophils) in the sample. For each sample, the percentage of NETting neutrophils was calculated in relation to the known total neutrophil number from which each NET sample obtained.

## Data Availability

The data that support the findings of this study are available from the corresponding author upon reasonable request.
