# Supplementary material for: The impact of neutrophil extracellular trap from patients with systemic lupus erythematosus on the viability, CD11b expression and oxidative burst of healthy neutrophils
Source: BMC Immunol. 2021 Feb 5;22:12. doi: 10.1186/s12865-021-00402-2 (PMC7863477; doi:10.1186/s12865-021-00402-2)
Supplement: Supplementary file 1 — Additional file 1. Supplementary Document 1. [file 12865_2021_402_MOESM1_ESM.docx]

**Supplementary document 1**

**Title Page**

**Title:** The impact of neutrophil extracellular trap from patients with systemic lupus erythematosus on the viability, activation and oxidative burst of healthy neutrophils.

**Author and Affiliation:**

1. Alimohammad Fatemi

Associate Professor of Rheumatology, Rheumatology Section, Department of Internal Medicine, School of Medicine, Isfahan University of Medical Sciences, Isfahan, Iran. E-mail: [a_fatemi@med.mui.ac.ir](mailto:a_fatemi@med.mui.ac.ir)

1. Razieh Alipour

PhD of Immunology, Immunology Department, Medical School, Isfahan University of Medical Sciences, Isfahan, Iran. E-mail: [ra_alipour@resident.mui.ac.ir](mailto:ra_alipour@resident.mui.ac.ir)

1. Hossein Khanahmad

Associate Professor of Medical Biotechnology, Department of Genetics and Molecular Biology, School of Medicine, Pediatric Inherited Diseases Research Center, Research Institute for Primordial Prevention of Non-communicable Disease, Isfahan University of Medical Sciences, Isfahan, Iran. E-mail: hossein_khanahmad@yahoo.com

1. Fereshteh Alsahebfosul

PhD, Associate Professor of Immunology, Immunology Department, Medical School, Isfahan University of Medical Sciences, Isfahan, Iran. E-mail: [alsahebfosoul@med.mui.ac.ir](mailto:alsahebfosoul@med.mui.ac.ir)

1. Alireza Andalib

PhD, Professor of Immunology, Immunology Department, Isfahan University of Medical Sciences, Isfahan, Iran. E-mail: [andalib@med.mui.ac.ir](mailto:andalib@med.mui.ac.ir)

1. Abbasali Pourazar (**Corresponding author**)

Professor of Immunohematology PhD, Department of Immunology, Medical School, Isfahan University of Medical Sciences, Hezar Jerib Street, Isfahan, Postcode: 81746-73695, I.R.IRAN. Tell: 09831379229031. E-mail: [pourazar@med.mui.ac.ir](mailto:pourazar@med.mui.ac.ir)

This document includes data of the participants. Also, a schematic diagram of treatment is included.

**Patients and controls**

Table S1 shows the characteristics of the SLE patients. Blood was taken from 17 healthy individuals as controls (14 women and three men) with a mean age of 33.23 years (Standard deviation (SD) = 9.18; range = 21-49 years ).

**Table S 1. The characteristics of the patients.**

| Patient  Number | Gender | Disease duration (year) | SLEDAI |
| --- | --- | --- | --- |
|  |  |  |  |
| 1 | F | 4 | 16 |
| 2 | F | 1 | 6 |
| 3 | F | 9 | 6 |
| 4 | F | NR | NR |
| 5 | F | 1 | 6 |
| 6 | F | 5 | 14 |
| 7 | F | 4 | 14 |
| 8 | F | 1 | 12 |
| 9 | F | New case | 8 |
| 10 | F | 12 | 9 |
| 11 | F | 20 | 4 |
| 12 | M | 1 | 8 |
| 13 | M | 14 | 10 |
| 14 | F | 12 | 6 |
| 15 | F | 5 | 2 |
| 16 | F | 17 | 14 |
| 17 | F | 1 | 9 |
| Mean ± SD | | 7.13 ± 6.42 | 9.00 ± 4.05 |

The mean age of the patients was 32.47 years (SD = 10.66 years; range = 18-51). F: female; M: male; NR: Not reported. SLEDAI: Systemic lupus erythematosus disease activity index.

**Treatments**

The types of treatment and control are schematically presented.

**Figure S1**

**A schematic diagram of the neutrophil culture**.

The schematic diagram of the treatment of neutrophils is shown in black in the figure and the subsequent stimulation related to “CD11b expression assay” is presented. Number (7) denotes the baseline expression. Numbers (6) and (5) are controls for the NET isolation; they determine whether the observed effects of the collected NET are actually from NET. Number (4( denotes whether NET (a mixture of the patient and healthy NETs) can change CD11b expression by itself. NCM: NETs’ control medium.
