# Supplementary material for: The impact of neutrophil extracellular trap from patients with systemic lupus erythematosus on the viability, CD11b expression and oxidative burst of healthy neutrophils
Source: BMC Immunol. 2021 Feb 5;22:12. doi: 10.1186/s12865-021-00402-2 (PMC7863477; doi:10.1186/s12865-021-00402-2)
Supplement: Supplementary file 2 — Additional file 2. Supplementary Document 2. [file 12865_2021_402_MOESM2_ESM.docx]

**Supplementary Document 2**

**Title:** The impact of neutrophil extracellular trap from patients with systemic lupus erythematosus on the viability, CD11b expression and oxidative burst of healthy neutrophils.

**Author and Affiliation:**

1. Alimohammad Fatemi

Associate Professor of Rheumatology, Rheumatology Section, Department of Internal Medicine, School of Medicine, Isfahan University of Medical Sciences, Isfahan, Iran. E-mail: [a_fatemi@med.mui.ac.ir](mailto:a_fatemi@med.mui.ac.ir)

1. Razieh Alipour

PhD of Immunology, Immunology Department, Medical School, Isfahan University of Medical Sciences, Isfahan, Iran. E-mail: [ra_alipour@resident.mui.ac.ir](mailto:ra_alipour@resident.mui.ac.ir)

1. Hossein Khanahmad

Associate Professor of Medical Biotechnology, Department of Genetics and Molecular Biology, School of Medicine, Pediatric Inherited Diseases Research Center, Research Institute for Primordial Prevention of Non-communicable Disease, Isfahan University of Medical Sciences, Isfahan, Iran. E-mail: hossein_khanahmad@yahoo.com

1. Fereshteh Alsahebfosul

PhD, Associate Professor of Immunology, Immunology Department, Medical School, Isfahan University of Medical Sciences, Isfahan, Iran. E-mail: [alsahebfosoul@med.mui.ac.ir](mailto:alsahebfosoul@med.mui.ac.ir)

1. Alireza Andalib

PhD, Professor of Immunology, Immunology Department, Isfahan University of Medical Sciences, Isfahan, Iran. E-mail: [andalib@med.mui.ac.ir](mailto:andalib@med.mui.ac.ir)

1. Abbasali Pourazar (**Corresponding author**)

Professor of Immunohematology PhD, Department of Immunology, Medical School, Isfahan University of Medical Sciences, Hezar Jerib Street, Isfahan, Postcode: 81746-73695, I.R.IRAN. Tell: 09831379229031. E-mail: [pourazar@med.mui.ac.ir](mailto:pourazar@med.mui.ac.ir)

This document includes the data on NET quantification and also comparisons between the patients and control individuals.

**Evaluation and Comparison of NET samples**

**Introduction**

Despite a large volume of publications on NET, a standard method for objectively and accurately quantifying it has not been established yet because of its inherent properties and some empirical limitations (1, 2). To date, many approaches, including computational and non-computational methods, have been suggested for NET quantification (2-4). NET is a mixture of DNA and proteins (5), however, in previous works, mostly assessment of DNA in NET samples was used (6-8). Determining the percentage of neutrophils forming NET, more commonly via a microscopic evaluation (9-11) or less frequently through DNA quantification (10, 12, 13) is another general method for expressing the extent of NET formation, but both approaches have their drawbacks (3, 4, 10). Because of the lack of a current ‘gold standard’ method of NET quantification, it is recommended that researchers utilize a method that is more appropriate for the specific study (4, 7). In the present study, we assayed both DNA and proteins in the isolated NET samples and also determined the percentage of NET-forming neutrophils by quantitative PCR (qPCR), which is a widely used method of DNA quantification with high sensitivity (14).

**Methods**

**Protein Quantification:** We determined the protein content of the isolated NET samples using the Bradford method. The Bradford reagent was prepared according to the standard protocol. In brief, 10 mg of Coomassie brilliant blue G-250 (*Sigma-Aldrich*) was dissolved in 5 ml 96% ethanol; then, 10 ml 85% phosphoric acid (*Merck*) was added. Of the solution, 5 ml was diluted to 50 ml with distilled water and was filtered through a grade 1 Whatman Filter paper immediately before the assay. RPMI medium without proteins was used as the blank sample and a serial dilution of bovine serum albumin (BSA) which was prepared by adding appropriate volumes of RPMI medium to a 1 mg/ml BSA source solution (BSA in PBS), was used as standard samples. The experiment was performed in a 96-well microplate using 10 μl of each blank, standard, or NET sample, and 200 μl of the Bradford reagent. All samples were prepared in triplicate, and the absorbance values were measured at 630 nm by ELISA^^[[1]](#footnote-1)^^ reader after 15 mins incubation at RT. To create a standard curve from values obtained for standard samples, Excel Software (Microsoft Office 2013) was used. The protein concentration of NET samples was determined using the resulting equation of the standard curve.

**DNA visualization:** We visualized the DNA content isolated from the NET samples on a gel; 10 µl of concentrated NET samples were loaded on 1% agarose gel and run at 90 V for about 40 mins and then visualization was performed using the gel doc system (ATP Gel Documentation, SUV).

**DNA Quantification:** DNA concentration was determined with two of the three generally used DNA quantification methods. Specifically, we assessed the concentration of DNA in concentrated NET samples by calculating UV absorbance ratios at OD_260_/OD_280_ and OD_260_/OD_230_ for all samples by a NanoDrop instrument.

DNA quantification of all concentrated NET samples was also carried out by qPCR targeting the genome sequence of human *TLR*4 (115 bp) using 2X Real-Time PCR Master Mix (BioFact™). PCR was performed with a 10 μl reaction mixture containing 5 μl SYBR Premix, 1 μl primers (0.5 μl of each Forward and Reverse primer), and 3.5 μl sterile purified water with 0.5 μl NET sample. Then, the shuttle PCR protocol was performed as follows: 15 min at 95 °C for enzyme activation, denaturation for 15 seconds at 95° C, annealing at 60°C for 30 seconds, and extension at 60°C for 30 seconds, for approximately 45 cycles. A serial dilution of the PCR product of the *TLR*4 gene of human DNA with known copy numbers of the gene was used for the preparation of the standard curve. All tests were assayed in triplicate. The sequence of the pair of primers that were used in qPCR is shown in Table S2.

Table S2. The sequence of TLR-4 primers that were used in qPCR.

| **Gene** | **Primer Sequence** | **Reference** |
| --- | --- | --- |
| TLR-4 | Forward: 5’TGGTGGAAGTTGAACGAATGG3’  Reverse: 5’AGGACCGACACACCAATGATG3’ | Diterich et al. (2003). Model of Borrelia-induced immunomodulation. |

Using the calculated copy numbers of the gene for each NET sample and the known initial number of neutrophils used for NET formation, we were able to estimate the percentage of netting neutrophils in all (SLE and control) samples.

**Results**

NET was collected by a non-enzymatic method as described in the main article (Methods section). Electrophoresis of the collected NET samples showed the large fragments of DNA in them (Figure S2).


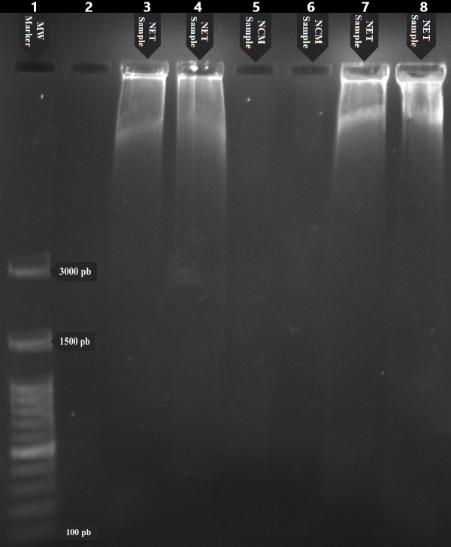


**Figure S2. 10 µm of the concentrated NET sample were loaded on agarose gel for the visualization of DNA.** Lane 1 corresponds to the molecular weight marker, with a higher band of 3000 bp and a lower 100-bp band. The high molecular weight bands in lanes 3, 4, 7, and 8 correspond to DNA present in NET samples. Two NCM (NET control medium) samples -collected from unstimulated neutrophils in NET-inducing experiments- were loaded in lanes 5 and 6. No sample was loaded in lane 2.

DNA concentration of NET samples was determined and compared between patients and controls. The amount of protein was also assessed and compared. For both DNA and protein, the increase in their amounts in patient samples compared to healthy samples was not statistically significant (Table S3).

**Table S3. The amount of DNA and protein in the collected NET samples**.

| *P_value_* | Protein (µg/ml) | Group | DNA (ng/ml) | *P_value_* |
| --- | --- | --- | --- | --- |
| 0.46 | 48.84 **±** 1.88 | **NET SLE** | 3316.24 **±** 402.33 | 0.63 |
|  | 43.90 **±** 1.98 | **NET Normal** | 2738.99 **±** 450.72 |  |
| 0.95 | 10.85 **±** 5.50 | **SLE NCM** | 0.4965 **±** 0.130 | 0.99 |
|  | 13.82 **±** 1.96 | **Normal NCM** | 0.584 **±** 0.238 |  |
| 0.00 | ***P_value_*** | | | 0.024 |

NCM: NET control medium; the medium collected from the unstimulated neutrophils in the NET-inducing experiments

Using qPCR, we evaluated the absolute number of NETting neutrophils in each sample; then, the percentage of NETting neutrophils was calculated in relation to the total number of neutrophils from which NET was collected, (Figure S3). For this purpose, the absolute copy number of a genomic DNA sequence (in this case a 115 bps sequence of *TLR*-4 gene) in all samples was obtained by drawing a standard curve; this number was assumed as the number of neutrophils that released their nuclear DNA (or neutrophils which formed NET) in each sample. For each sample, the percentage of NETting neutrophils was calculated in relation to the known total neutrophil number from which each NET sample was obtained.

The comparison of the percentage of NETting neutrophils between the two groups showed an increase in the patient group that was not statistically significant (Table S4).


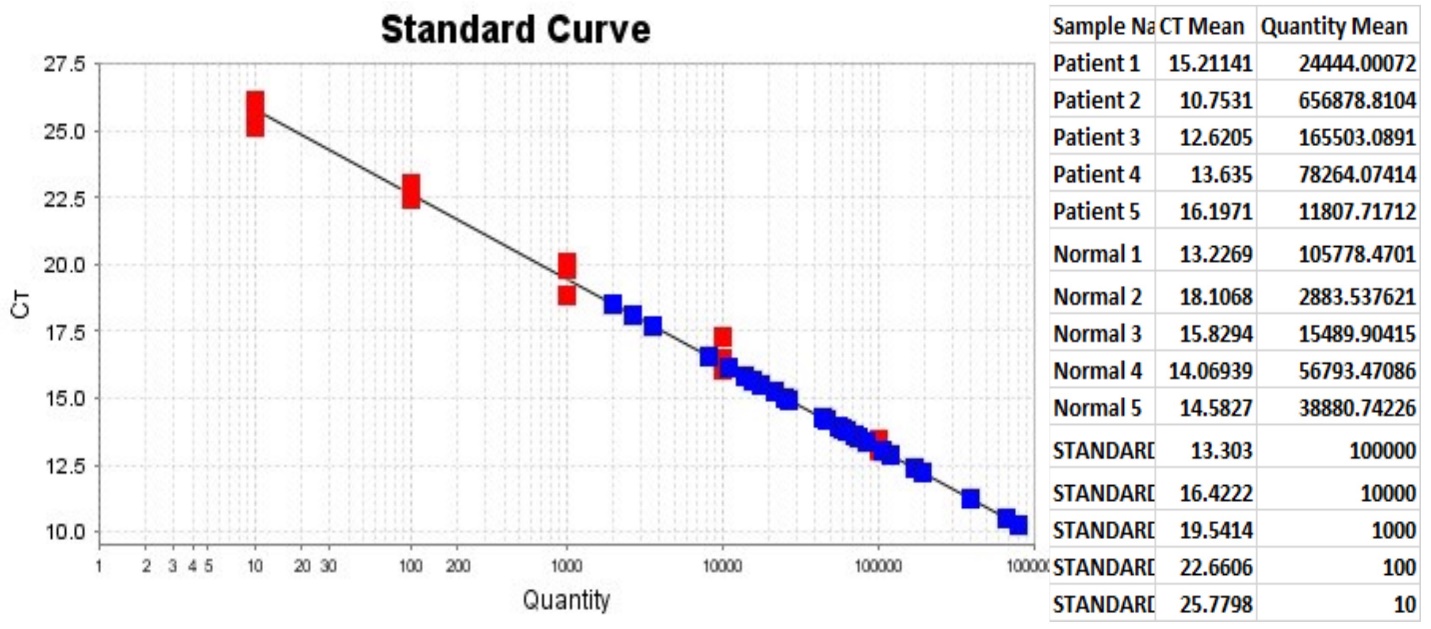


**Figure S3.A standard curve and its corresponding brief data plotted using StepOne™ software.** The number of NETting neutrophils (or the copy number of the target genomic sequence) in 10 samples (5 SLE patients and 5 controls) is shown. With the standard curve generated by data from the standard dilution series, the software determined the absolute quantity for each sample. In the plot, red squares correspond to standards, and blue squares correspond to samples (all tests were performed in triplicate). The quantity of a sample refers to the absolute copy number of the target gene (here, TLR-4) in the sample, which was taken as the number of neutrophils that released their nuclear DNA (or the absolute count of NETting neutrophils) in the sample. For each sample, the percentage of NETting neutrophils was calculated in relation to the known total neutrophil number from which each NET sample obtained.

Table S4. The percentage of NETting neutrophils.

| **Group** | **Patient** | **Control** |
| --- | --- | --- |
| **NET** **Percentage** | 36.66 **±** 6.51 | 32.43 **±** 9.33 |
| *P_value_* | 0.704 | |

**Discussion**

Recently [Wirestam](https://www.frontiersin.org/people/u/737082) et al. (15), have reviewed a lot of previous reports on SLE neutrophils and highlighted that in patients suffering from SLE, neutrophils display an activated phenotype and form NET more readily than neutrophils of healthy individuals but in the present study, we could not find a statistically significant difference between the NET-forming ability of neutrophils from healthy controls and SLE patients after PMA stimulation. However, PMA is a strong stimulus of NET production (16), and it is possible that the intensive activation by PMA covered the difference between the two neutrophil groups in terms of their NET-releasing ability. It should be noted that in previous studies the difference between SLE and normal neutrophils in producing NET was shown when neutrophils were stimulated by stimuli other than PMA. For example, although Garcia-Romo et al. (17) stimulated neutrophils with PMA and anti-RNP^^[[2]](#footnote-2)^^ IgG but reported a higher NET formation of lupus neutrophils compared to healthy neutrophils only in the group stimulated by anti-RNP IgG.

**References**

1. Masuda S, Nakazawa D, Shida H, Miyoshi A, Kusunoki Y, Tomaru U, et al. NETosis markers: quest for specific, objective, and quantitative markers. Clinica chimica acta. 2016;459:89-93.

2. van Breda SV, Vokalova L, Neugebauer C, Rossi SW, Hahn S, Hasler P. Computational Methodologies for the in vitro and in situ Quantification of Neutrophil Extracellular Traps. Frontiers in Immunology. 2019;10:1562.

3. Gavillet M, Martinod K, Renella R, Harris C, Shapiro NI, Wagner DD, et al. Flow cytometric assay for direct quantification of neutrophil extracellular traps in blood samples. American journal of hematology. 2015;90(12):1155-8.

4. Naccache PH, Fernandes MJ. Challenges in the characterization of neutrophil extracellular traps: The truth is in the details. European journal of immunology. 2016;46(1):52-5.

5. Liew PX, Kubes P. The neutrophil’s role during health and disease. Physiological reviews. 2019;99(2):1223-48.

6. Barrera-Vargas A, Gómez-Martín D, Carmona-Rivera C, Merayo-Chalico J, Torres-Ruiz J, Manna Z, et al. Differential ubiquitination in NETs regulates macrophage responses in systemic lupus erythematosus. Annals of the rheumatic diseases. 2018;77(6):944-50.

7. Saffarzadeh M, Juenemann C, Queisser MA, Lochnit G, Barreto G, Galuska SP, et al. Neutrophil extracellular traps directly induce epithelial and endothelial cell death: a predominant role of histones. PloS one. 2012;7(2):e32366.

8. Kambas K, Mitroulis I, Apostolidou E, Girod A, Chrysanthopoulou A, Pneumatikos I, et al. Autophagy mediates the delivery of thrombogenic tissue factor to neutrophil extracellular traps in human sepsis. PloS one. 2012;7(9):e45427.

9. Remijsen Q, Berghe TV, Wirawan E, Asselbergh B, Parthoens E, De Rycke R, et al. Neutrophil extracellular trap cell death requires both autophagy and superoxide generation. Cell research. 2011;21(2):290.

10. von Köckritz-Blickwede M, Chow O, Ghochani M, Nizet V. 7-visualization and functional evaluation of phagocyte extracellular traps. Immunology of Infection. 2010;3.

11. Yu Y, Koehn C, Yue Y, Li S, Thiele GM, Hearth-Holmes MP, et al. Celastrol inhibits inflammatory stimuli-induced neutrophil extracellular trap formation. Current molecular medicine. 2015;15(4):401-10.

12. Kahlenberg JM, Carmona-Rivera C, Smith CK, Kaplan MJ. Neutrophil extracellular trap–associated protein activation of the NLRP3 inflammasome is enhanced in lupus macrophages. The journal of immunology. 2013;190(3):1217-26.

13. Lim MBH, Kuiper JW, Katchky A, Goldberg H, Glogauer M. Rac2 is required for the formation of neutrophil extracellular traps. Journal of leukocyte biology. 2011;90(4):771-6.

14. Nakayama Y, Yamaguchi H, Einaga N, Esumi M. Pitfalls of DNA quantification using DNA-binding fluorescent dyes and suggested solutions. PLoS One. 2016;11(3):e0150528.

15. Wirestam L, Arve S, Linge P, Bengtsson AA. Neutrophils-important communicators in systemic lupus erythematosus and antiphospholipid syndrome. Frontiers in immunology. 2019;10:2734.

16. Petretto A, Bruschi M, Pratesi F, Croia C, Candiano G, Ghiggeri G, et al. Neutrophil extracellular traps (NET) induced by different stimuli: A comparative proteomic analysis. PloS one. 2019;14(7).

17. Garcia-Romo GS, Caielli S, Vega B, Connolly J, Allantaz F, Xu Z, et al. Netting neutrophils are major inducers of type I IFN production in pediatric systemic lupus erythematosus. Science translational medicine. 2011;3(73):73ra20-73ra20.

1. [↑](#footnote-ref-1)
2. anti-ribonucleoprotein [↑](#footnote-ref-2)
