# Supplementary figures and images for: The impact of neutrophil extracellular trap from patients with systemic lupus erythematosus on the viability, CD11b expression and oxidative burst of healthy neutrophils
Source: BMC Immunol. 2021 Feb 5;22:12. doi: 10.1186/s12865-021-00402-2 (PMC7863477; doi:10.1186/s12865-021-00402-2)

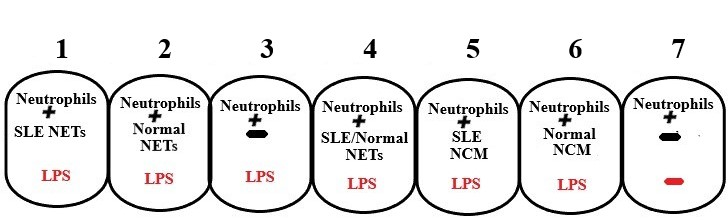

Supplement: Supplementary file 3 — Additional file 3: Fig. S1 A schematic diagram of the neutrophil culture. The schematic diagram of the treatment of neutrophils is shown in the figure in black and the subsequent stimulation related to “CD11b expression assay” is presented. Number (7) denotes the baseline expression. Numbers (6) and (5) are controls for the NET isolation; they determine whether the observed effects of the collected NET are really from NET. Number (4) determines whether NET (a mixture of the patient and healthy NET) can change CD11b expression by itself. NCM: NETs’ control medium. [file 12865_2021_402_MOESM3_ESM.tiff]

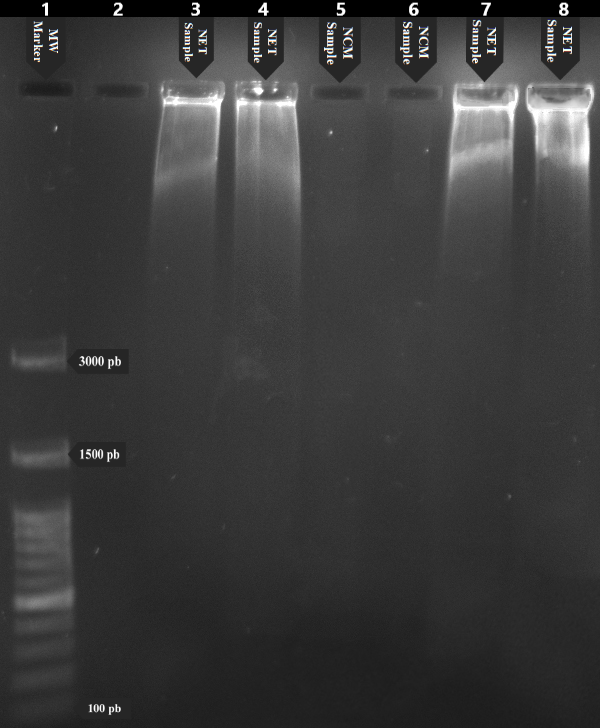

Supplement: Supplementary file 4 — Additional file 4: Fig. S2 The visualization of DNA of NET in gel. Lane 1 corresponds to the molecular weight marker, with a higher band of 3000 bp and a lower 100-bp band. The high molecular weight bands in lanes 3, 4, 7, and 8 correspond to DNA present in NET samples. Two NCM (NET control medium) samples -collected from unstimulated neutrophils in NET-inducing experiments- were loaded in lanes 5 and 6. No sample was loaded in lane 2. [file 12865_2021_402_MOESM4_ESM.tiff]

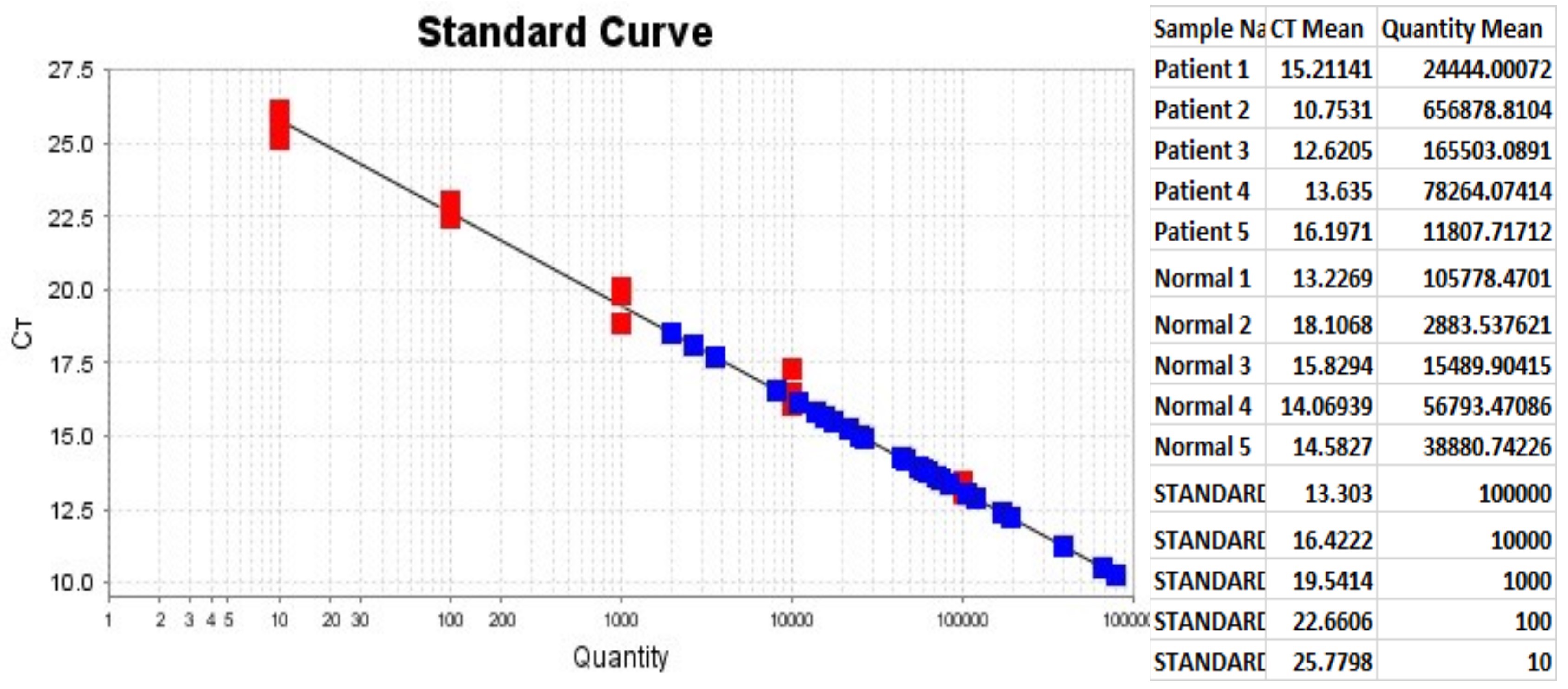

Supplement: Supplementary file 5 — Additional file 5: Fig. S3 A standard curve and its corresponding brief data plotted using StepOne™ software. The number of NETting neutrophils (or the copy number of the target genomic sequence) in 10 samples (5 SLE patients and 5 controls) is shown. With the standard curve generated by data from the standard dilution series, the software determined the absolute quantity for each sample. In the plot, red squares correspond to standards, and blue squares correspond to samples (all tests were performed in triplicate). The quantity of a sample refers to the absolute copy number of the target gene (here, TLR-4) in the sample, which was taken as the number of neutrophils that released their nuclear DNA (or the absolute count of NETting neutrophils) in the sample. For each sample, the percentage of NETting neutrophils was calculated in relation to the known total neutrophil number from which each NET sample obtained. [file 12865_2021_402_MOESM5_ESM.jpg]
